# Different ways to present clinical cases in a classroom: video projection versus live representation of a simulated clinical scene with actors

**DOI:** 10.1186/s12909-019-1494-1

**Published:** 2019-03-04

**Authors:** M. J. Robles, Ramón Miralles, Ascension Esperanza, Mercedes Riera

**Affiliations:** 1 Geriatric Department Consorcio Parc de Salut Mar de Barcelona, Paseo Marítimo, 25-29, 08003 Barcelona, Spain; 2 Faculty of Medicine, Universidad Autónoma de Barcelona, Barcelona, Spain; 3 Department of Nursing, Consorcio Parc de Salut Mar de Barcelona, Barcelona, Spain

## Abstract

**Background:**

Use of the video digital format in the classroom is a common way to present clinical cases to stimulate discussion and increase learning. A simulated live performance with actors, also in the classroom, could be an alternative way to present cases that may be more attractive to arouse students’ interest and attention. The aim of the present study was to compare the learning process between a group of students who saw a clinical case as a simulated live scene in the classroom and others seeing the same clinical case projected by video.

**Method:**

One hundred and thirty-one students (69 from physiotherapy and 62 from medicine) attended an interactive seminar on delirium in older people. Each group was subdivided into two groups: one saw the clinical case as a theatrical performance in the classroom (scene group; *n* = 68), while the other saw the same case projected on video (video group; *n* = 63). Before and after attending the seminar, students answered a questionnaire [four questions on theoretical knowledge of delirium (score 0–7) and two on subjective learning perception (linear scale: 0–10) (score 0–20)]. At the end, a further question was included on the usefulness of the scene or a video in the learning process (linear scale: 0–10).

**Results:**

Students in both groups (live scene and video) significantly improved in all questionnaire scores after the seminar (*p* = 0.001) with a large Effect Size (ES > 0.80). Students of the scene group obtained higher scores on theoretical delirium knowledge [6.41 ± 0.73 vs 5.93 ± 1.31 (*p* = 0.05)], subjective learning perception questions (what they thought they knew about delirium) (16.28 ± 3.51 versus 15.92 ± 2.47 (*p* = 0.072)], and the overall questionnaire (22.45 ± 4.15 versus 21.48 ± 2.94 (*p* = 0.027)] than the video group. Students of the scene group opined that live scene was very useful for learning with a mean score of 9.04 ± 1.16 (range 0–10), and opinion in the student’s video group scored 8.21 ± 1.22 (*p* = 0.001).

**Conclusions:**

All students improved significantly their knowledge but those who saw the theatrical performance obtained slightly better results, which suggest that this form of clinical case presentation in the classroom may be an alternative at least as effective as traditional video projections.

## Background

The use of images recorded in video format is widespread in the field of training and teaching in health science [1]. Modern digital cameras and the possibility to recording videos easily with a mobile phone have facilitated the use of images and videos that often accompany theoretical lessons and lectures presented in the classroom. Moreover, it is well known that clinical cases filmed in video digital format can be a key tool to learning and illustrating real life problems from that encourage discussion on clinically significant aspects and improve reflective approaches concerning values, attitudes and beliefs [1, 2]. However, in recent years, the overuse of video may have become in itself overly routine with a less incentive capacity to maintain students’ attention. To show in the classroom the same case that could be projected in video, in the form of a live simulated scene with actors as a theatrical performance, can also be an attractive way to present a clinical situation for discussion. The use of simulated patients has proved to be a useful learning method in health science education. Its value as a teaching tool usually requires students to face the simulated patient individually and undertake an activity (e.g. a medical history aimed at a problem or a physical examination) [3]. This model is complex to organise and difficult to carry out in the context of routine academic activity.

Some authors have suggested that students can also learn by watching the simulation without participating actively in it −the concept of learning by observing− which has become popular in the international literature under the term “vicarious learning” or “learning by seeing others” [4, 5]. This concept offers a theoretical and practical basis for carrying out simulation techniques in conventional classrooms, which, has the advantage that all students can observe the simulation at the same time with the appropriate feedback and discussion (debriefing). Vicarious learning occurs both if the clinical case is seen by students in the form of a video projection or as a live simulated scene with actors (as if they were theatregoers). Video has the advantage that it can be re-played as many times as necessary, while repeating the scene “live” is more complicated and requires actors having to return to the classroom. Such difficulties could be recompensed if the impact of seeing the scene “live” leads to better learning. Previous experience performed in our setting showed that the use of actors in the classroom to simulate a clinical scenario may per se be a powerful stimulus to arouse students’ interest and attention and break the monotony of the classroom [6].

The aim of the present study was to ascertain whether differences existed in learning between the group of students who saw a simulated clinical scene with actors in the classroom (theatrical performance) and the other who saw the same case in a video projection.

## Methods

A teaching seminar was conducted at the Parc Salut Mar, a university hospital in Barcelona (Spain). Sixty-nine students from physiotherapy and sixty-two from medicine, from two universities (Universidad Autónoma de Barcelona and Universidad Pompeu Fabra) attended the seminar. This seminar was based on the approach to a clinical problem in the form of a simulated live scene with actors in the classroom or projected by video; this was followed by an interactive presentation in the same classroom, which was the same for both options. The students of each program attended the seminar separately in different days; the students of each program were divided into reduced groups chosen at random (the students from both programs were they kept separate).

**Fig. 1:**
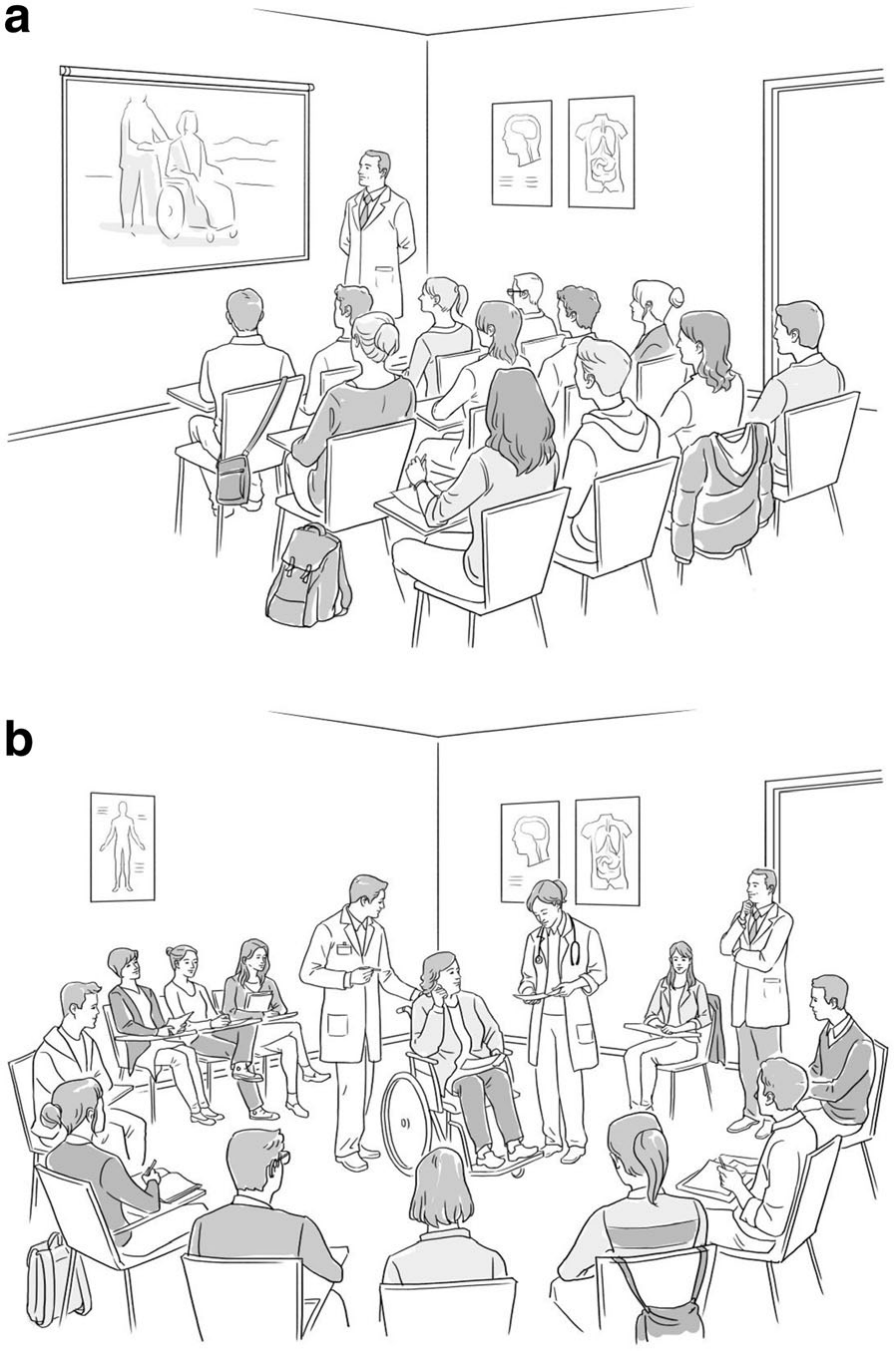
*(The drawings depicted have been created on behalf of the authors, and copyright have been paid, and written permission to use it in a medical journal has also been given by the artist (Ferreiro Iglesias Studio SL, Igualada, Barcelona)* **a**. The pre-recorded scene was displayed on a screen wall using the usual projector that was already installed in the classroom. **b**. Image that shows the live simulation in the centre of the classroom surrounded by the students

**Fig. 2:**
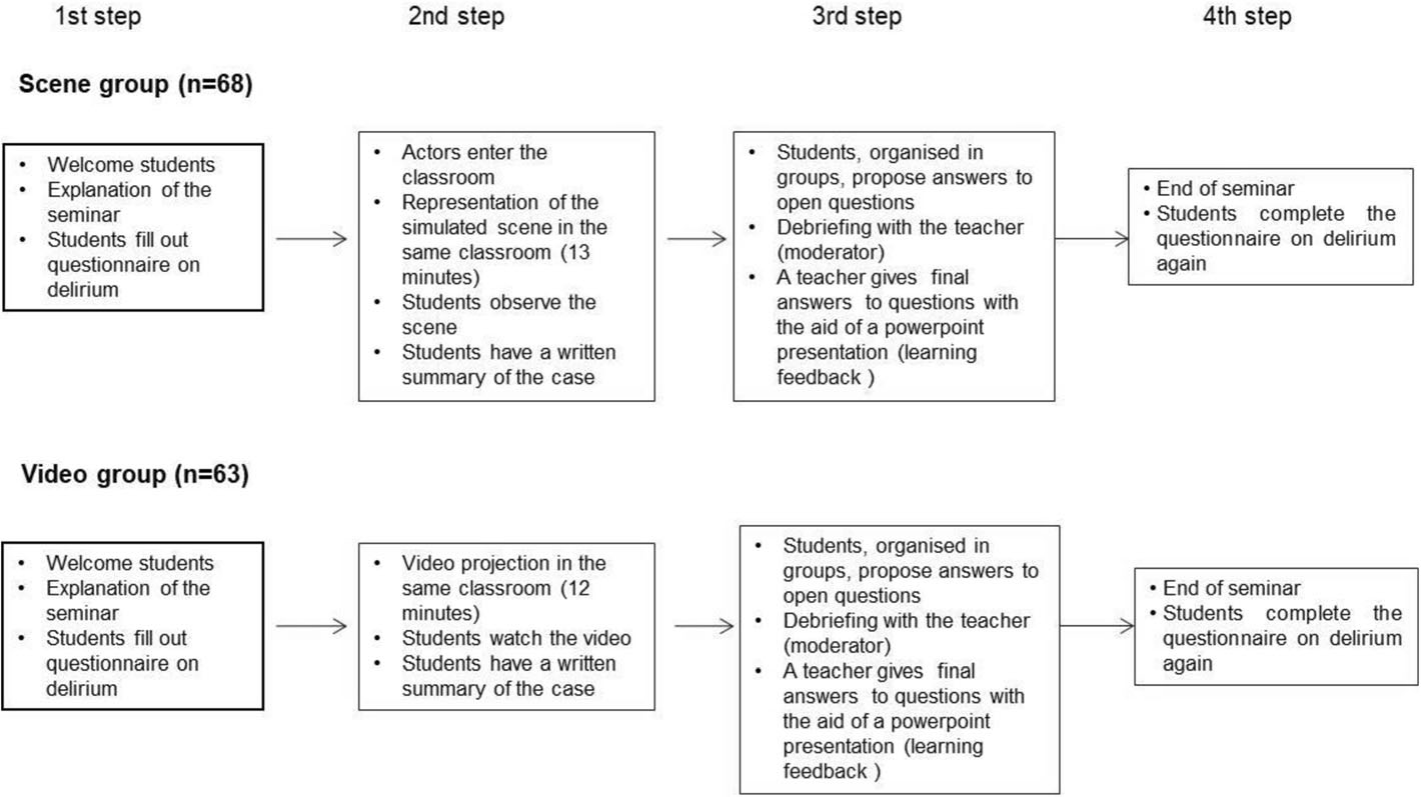
Diagrammatic representation of the seminar on delirium with a simulated clinical scenario in the classroom or with video projection (duration: 2 h). Groups attended the seminar separately

The seminar was performed four times, two in the form of a simulated live scene in the classroom with actors (one for the physiotherapy students and the other for the medicine students) (scene group; *n* = 68), and two with the projection of the case recorded on video which had been filmed previously (the same case and the same actors) (video group; *n* = 63) (for both groups of students, respectively). Students from the video group watched the pre-recorded scene all together in the classroom in order to preserve the same conditions as in the other group of students who saw the live performance. At the beginning of the seminar, students were informed of what was happen. They were provided with a written summary of the case to be simulated and answered a questionnaire with four theoretical questions related to delirium in the elderly, and two questions on students’ subjective learning perception with regard to what they thought they knew about delirium (see footnote of Table 1). The scene was then simulated live in the classroom as a theatrical performance for the scene group while the case was projected on a wall screen for the video group, after which open questions on the case were asked and discussed (debriefing). The students were divided into groups and encouraged to give their answers; the teacher then provided the correct answers using a powerpoint presentation (learning feedback). Students watched the scene or the video in the same classroom where lectures are usually given. The clinical problem represented was entitled “What’s wrong with my father?” and involved three actors. A brief summary of the clinical case and the two different ways in which it was performed are shown in Fig. 1. At the end of the seminar, students again answered the same questionnaire as at the beginning, but with a new question added on their opinion of the usefulness of the scene or video (depending on the case) for learning [Do you think the simulated patient scene or video as teaching tools were useful for understanding and treating the delirium syndrome? (range 0–10)]. An overall diagram of the seminar and details of the teaching activity are shown in Fig. 2. Questionnaires were later corrected by experts in geriatrics blinded to which group they belonged (scene or video). Correction criteria of theoretical questions had been previously agreed. According to what was considered important, an arbitrary score was assigned to each of the possible answers to the theoretical questions (see footnote in Table 1).

**Table 1:** Comparison of scores obtained on the questionnaires of knowledge about delirium before and after the seminar in both groups and between groups

	Scene group (*n* = 68)	Video group (*n* = 63)	Differences between groups (*p* value)
Total theoretical question score^a^ (range 0–7)*			
Before (mean ± SD)	3.51 ± 1.47	2.95 ± 1.50	0.036
After (mean ± SD)	6.41 ± 0.73	5.93 ± 1.31	0.050
Differences (mean ± SD)Ɨ	2.90 ± 1.15	2.98 ± 1.43	0.439
p. value (before vs after)	0.001	0.001	
Effect size (before vs after)ƗƗ	1.97	1.98	
Total subjective learning perception question score^a^ (range 0–20)**			
Before (mean ± SD)	10.54 ± 3.59	9.40 ± 3.70	0.108
After (mean ± SD)	16.28 ± 3.51	15.92 ± 2.47	0.072
Differences (mean ± SD)Ɨ	5.74 ± 4.61	6.51 ± 3.27	0.503
p. value (before vs after)	0.001	0.001	
Effect size (before vs after)ƗƗ	1.59	1.76	
Total score (sum of all questions from 1 to 6)^a^ (range: 0–27 points)			
Before (mean ± SD)	14.05 ± 4.38	12.35 ± 4.56	0.044
After (mean ± SD)	22.45 ± 4.15	21.48 ± 2.94	0.027
Differences (mean ± SD)Ɨ	8.40 ± 4.86	9.48 ± 5.03	0.682
p. value (before vs after)	0.001	0.001	
Effect size (before vs after)ƗƗ	1.91	2.08	

*Four theoretical questions about delirium; definition (range 0–2 points), describe predisposing factors (range 0–1 point), describe precipitating factors (range 0–2 points) and make a list of measures to improve and prevent progression of delirium (range 0–2 points)

**Two subjective learning perception questions [to what degree would you be able to detect the risk of a confusional syndrome? (Linear scale from 0 to 10 points) and to what degree would you be able to advise a plan of interventions to prevent delirium in an elderly patient? (Linear scale from 0 to 10 points)]

^a^Complete questionnaire can be obtained in the supplementary appendix S1 of a previous publication [6]

Ɨ Average increase in questionnaire score after the seminar

ƗƗ > 0.80 signifies large change

### Assessment of results and statistical analysis

Students’ results (both on theoretical and subjective knowledge) and their opinions on teaching activity were collected from the questionnaires in the form of numerical scores. These were treated as quantitative variables (ordinals) and their results are expressed as mean and standard deviation. Student’s t-test or Mann-Whitney U test were used to compare scores on the questionnaires (mean comparison of different groups), depending on whether the variables followed a normal distribution. In the case of comparison of means in the same group (before and after), Student’s t-test was used for paired data and also Wilcoxon’s t-test depending on the type of variable distribution. Standard categorisation of effect size (ES) was applied to ascertain the magnitude of change scores on the questionnaires before and after the seminar, according to the formula of Cohen in which ES is equal to the difference between mean scores on a question at the beginning and end of the seminar, divided by the standard deviation of the mean of scores obtained at baseline. This calculation converts the change score into a standard unit of measurement which evaluates whether the difference between the mean score of a question before and after the seminar is small or large depending on the number of standard deviations separating them. The guidelines define an ES of 0.20 as small, 0.50 as moderate and 0.80 as large [7, 8]. Finally, a statistically-significant difference was considered when *p* value was < 0.05.

## Results

The mean scores obtained by students on the questionnaires on understanding of delirium before and after the seminars in both groups (one with the simulated clinical live scene and the other with the projection of the same case in a digital video) are shown in Table 1. In both groups, all questionnaire scores showed a statistically-significant increase after the seminar with a large ES (> 0.80). Students who attended the seminar in the scene group obtained higher scores on theoretical delirium knowledge, subjective learning perception questions (what they thought they knew about delirium) and the overall questionnaire than the video group. The difference was only statistically significant in the latter. Students in the scene group returned a mean score on the question of opinion of 9.04 ± 1.16 (in this question, the student assessed the usefulness of the simulated clinical scene or the video projection) (see Methods). This score was 8.21 ± 1.22 in the video group (*p* = 0.001).

## Discussion

The present study shows that all students both the scene group and the video group significantly improved their knowledge of the main topic. This change was significant from a practical point of view (large ES > 0.80). Thus, we can affirm that the seminar was effective in both groups of students.

Similarly, in this study, a tendency towards slightly better results was observed in students who saw the live scene in the classroom than in those who saw the case by video projection. The format of the seminar and the questions discussed on the clinical case (debriefing) and the information provided by the powerpoint were the same in both groups (scene and video); this suggests that the small difference observed could be attributed to the different format in which the clinical case was presented. These results could be in line with those of other authors who published positive teaching experiences utilising actors to represent a clinical scene in the classroom as if it were a theatre [9, 10]. Ünalan et al. demonstrated that a theatrical performance on a lecture about headache followed by debriefing about the symptoms, signs and differential diagnosis was very useful for students’ learning. In that experience, the authors stated that over 90% of students opined that the theatre made it easier to understand the topic [9]. We found few studies in the literature in which both forms of clinical case presentation were compared in conditions similar to ours. Therefore, it is difficult to contrast our results with other published experiences. Hernandez et at [11], used medical simulation to help teaching basic pharmacology in a manner that can more easily integrate with clinical sciences; the simulation involved the use of both pre-recorded and live streaming simulated scenes in a clinical vignette format. Alqahtani et al. conducted a study which aimed to assess the efficacy of procedural video compared to a live demonstration in transferring skills to four-year undergraduate dental students during a laboratory session. The authors concluded that procedural video was equally as effective as a live demonstration [12]. Aghababaeian et al. studied 144 emergency personnel randomly classified in two groups: the first group used an educational video method and the second a role-playing method to learn and perform medical emergencies. Those authors observed that no significant difference existed between the two training methods in performance and immediate knowledge, as in our study; however a statistical advantage was observed for the role-playing method in lasting performance when the same students were re-evaluated after 15 days, suggesting that the latter method encouraged longer and more lasting learning [13]. The experience of those authors and the fact that in our study the results of the clinical scene group were slightly better at the end of seminar suggest that the simulated “live performance” could facilitate better learning. This could be explained by the fact that the live scene would provide an element of greater authenticity (the theatre brings an element of realism that renders it unique in itself), ensuring that learning takes place in a more attractive context than the video format and enabling the students to retain the knowledge more effectively. Although this idea seems attractive, our results do not permit us to affirm that the learning in one group was better than in the other because the differences between the groups are small; thus, both forms of clinical case presentation are useful and each method has its own advantages and limitations. Definitely both the live scene and pre-recorded simulation case required a lot more preparation. But if the case is presented as a theatrical performance, the dramatization of a clinical vignette by faculty lets the audience experience and appreciate the case from a different perspective and in some instances theatre could help to teach empathy [11]. Some limitations in the live scene could be that actors must rehearse and be prepared for the unexpected that can go wrong when it is done live.

Even though the pre-recorded simulation case is also more dependent on technology and bandwidth strength needed for transmission of good quality visual and sound, after having the technology problems resolved, it can be an excellent tool to minimize the risk of something going wrong in the live scene, it allows watching the scene as many times as one wants, pause rewind, etc. Finally, in making decisions for which method to use other factors can be taken into account such as cost, feasibility and teacher preference among other things.

The present study had the limitations that the students were not randomised individually for their group assignment. The groups were distributed following the common university method (alphabetical order, compatibility of timetables and shifts, etc.), which may account for both groups not being similar in basal conditions. Before the seminar, students in the clinical scene group had better scores on the theoretical questionnaire than the video group. Although this difference was slight, it could have influenced the higher score obtained by this group at the end of the seminar.

On the other hand, we believe the results in learning of the clinical scene group would have been better if the live performance had been interactive, allowing the students to be involved at some point of the live performance by interacting with the actors, as in the experiences of other authors [10].

Finally, from our experience, lectures supported by clinical case presentation as a theatrical performance in a classroom appears to be an attractive alternative to the traditional format and as effective as a video projection. We advocated carrying out further similar experiences in the future to widen this learning practice for it to be better evaluated.

## Conclusions

All students both the scene group and the video group significantly improved their knowledge of the main topic, but the fact that the results of the clinical scene group were slightly better at the end of seminar suggest that the simulated “live performance” could facilitate better learning providing an element of greater authenticity, ensuring that learning takes place in a more attractive context than the video format and enabling the students to retain the knowledge more effectively. Lectures supported by clinical case presentation as a theatrical performance in a classroom appears to be an attractive alternative to the traditional format and as effective as a video projection.

## Abbreviations

AE: Ascensión Esperanza
ES: Effect Size
MJR: María José Robles
MR: Mercedes Riera
RM: Ramón MIralles
VS: versus

## References

[1] Pinsky LE, Wipf JE (2000). A picture is worth a thousand words: practical use of videotape in teaching. J Gen Intern Med.

[2] Hakkarainen P, Vapalahti K (2011). Meaningful learning through video-supported forum-theater. IJTLHE..

[3] Elley CR, Clinick T, Wong C, Arroll B, Kennelly J, Doerr H, Moir F, Fishman T, Moyes SA, Kerse N (2012). Effectiveness of simulated clinical teaching in general practice: randomised controlled trial. J Prim Health Care.

[4] Roberts D (2010). Vicarious learning: a review of the literature. Nurse Educ Pract.

[5] O’Regan S, Molloy E, Watterson L (2016). Observer roles that optimize learning in healthcare simulation education: a systematic review. Adv Simul.

[6] Robles MJ, Esperanza A, Pi-Figueras M, Riera M, Miralles R (2017). Simulation of a clinical delirium scenario with actresses in the classroom: a useful method of learning clinical delirium management. Eur Geriatr Med.

[7] Cohen J. Statistical power analysis for the behavioral sciences. 2nd ed. New York; Hillsdale, N.J.: L. Erlbaum Associates; 1988.

[8] Casado A, Prieto L, Alonso J (1999). El tamaño del efecto de la diferencia entre dos medias: ¿estadísticamente significativo o clínicamente relevante?. Med Clin (Barc).

[9] Ünalan PC, Uzuner A, Çifçili S, Akman M, Hancıoğlu S, Thulesius H (2009). Using theatre in education in a traditional lecture oriented medical curriculum. BMC Med Educ.

[10] Jacobsen T, Baerheim A, Lepp MR, Schei E (2006). Analysis of role-play in medical communication training using a theatrical device the fourth wall. BMC Med Educ..

[11] Hernandez M, Giannini J, Alston S, Vasauskas A (2017). Use of live-stream and pre-recorded simulation in the individual readiness assessment test during TBL, a novel approach. Med.Sci.Educ..

[12] Alqahtani ND, Al-Jewair T, Al-Moammar K, Albarakati SF, ALkofide EA (2015). Live demonstration versus procedural video: a comparison of two methods for teaching an orthodontic laboratory procedure. BMC Med Educ..

[13] Aghababaeian H, Sedaghat S, Tahery N, Moghaddam AS, Maniei M, Bahrami N, Ahvazi LAA (2013). Comparative study of the effect of triage training by role-playing and educational video on the knowledge and performance of emergency medical service staffs in Iran. Prehosp Disaster Med.

[14] Universitat Autònoma de Barcelona. Codi de bones pràctiques en la recerca. [Disponible en : https://www.uab.cat/doc/codibonespractiques_recerca (accedido el 29 de diciembre 2018)].

